# 

**Published:** 1918-09-14

**Authors:** 


### The Hospital

The Workers' Newspaper of Administrative Medicine and Institutional Life, Administration, National Insurance and Health.

CONTENTS.

The Nomenclature of Diseases ... ... ... 509
The Sacrifice of the Civilian... ... ... ... 510
Hospital and Institutional News ... ... ... 511
The King's Fund for the Disabled—The Disabled in Industry—Hospitals on the Army Model—The late Dr. Saundby: An Honourable Career—Death of a Medical Authoress—A Leicester Philanthropist—The Achievement of Charing Cross Hospital—The American Hospital Association and the War—The Larger Thanks—The Limit—The Future of Mechanical Diversions—The Cost of "Asylum Patients"—The Removal of German Wounded from Netley Hospital—A New Sanatorium for York?—Distinction for Mr. George Robey—A New Thing in Architecture—The "Misconduct Rules" of Approved Societies—Dutch Social Work in Connection With Nervous Disease—This Week's Drug Market—To Our Readers.
Papers on Madness ... ... ... ... ... 515
IV. The Sole Test of Sanity.
The Practitioner's Hobbies and Relaxations ... 517
Antique Hunting and its Philosophy.
Australian Notes ... ... ... ... ... ... 518
Doctors and Lodges: The Commission's Finding.
The Coal Shortage ... ... ... ... ... 519
Sir Guy Calthrop's Appeal to the Medical Profession.
The Notification of Ophthalmia Neonatorum in Scotland ... ... ... ... ... ... 519
The Nurse's Voice ... ... ... ... ... 520
The Power of the College of Nursing.
The Matrons' and Sisters' Department ... ... 521
Nursing Progress and its Developments: The Local Finance Committee.
Round the Hospitals ... ... ... ... ... 521
College of Nursing Developments—Local Centres, Newcastle and London—The Year's Work at Liverpool Royal Infirmary—Matrons, and Nurse Agitators—Gallantry in Hospital Ships—Gymnastics for Probationers.
The Editor's Letter-Box ... ... ... ... 525
Matrons, and Nurse Agitators.
The London Hospital Quarterly Court ... ... 525
The Problem of the Discharged Soldier.
Matrons' and Sisters' Appointments ... ... ... 526
Institutional Worker ... ... ... ... ... 1
Accommodation of Children in London Hospitals.
Special Rations for Invalids.
September Question.
Enquiries and Answers.

